# Automatization and improvement of μCT analysis for murine lung disease models using a deep learning approach

**DOI:** 10.1186/s12931-020-01370-8

**Published:** 2020-05-24

**Authors:** Gerald Birk, Marc Kästle, Cornelia Tilp, Birgit Stierstorfer, Stephan Klee

**Affiliations:** 1 Department of Drug Discovery Sciences, Boehringer Ingelheim Pharma GmbH & Co. KG, Biberach an der Riss, Germany; 2 Department of Immunology and Respiratory Disease Research, Boehringer Ingelheim Pharma GmbH & Co. KG, Biberach an der Riss, Germany

## Abstract

**Background:**

One of the main diagnostic tools for lung diseases in humans is computed tomography (CT). A miniaturized version, micro-CT (μCT) is utilized to examine small rodents including mice. However, fully automated threshold-based segmentation and subsequent quantification of severely damaged lungs requires visual inspection and manual correction.

**Methods:**

Here we demonstrate the use of densitometry on regions of interest (ROI) in automatically detected portions of the lung, thus avoiding the need for lung segmentation. Utilizing deep learning approaches, the middle part of the lung is found in a μCT-stack and a ROI is placed in the left and the right lobe.

**Results:**

The intensity values within the ROIs of the μCT images were collected and subsequently used for the calculation of different lung-related parameters, such as mean lung attenuation (MLA), mode, full width at half maximum (FWHM), and skewness. For validation, the densitometric approach was correlated with histological readouts (Ashcroft Score, Mean Linear Intercept).

**Conclusion:**

We here show an automated tool that allows rapid and in-depth analysis of μCT scans of different murine models of lung disease.

## Introduction

Computed tomography (CT) methodology is important in identifying pathological changes in various organs [1]. A number of studies have shown the value of using CT for diagnosis of lung diseases and outcome prediction of patients [2, 3]. While CT is a powerful tool, radiologists require time and experience to analyze CT data in detail [4]. The growing field of analysis automation (e.g. by deep learning (DL), neural networks) is helping to reduce efforts for CT analysis in general and in the field of lung diseases [5].

A number of animal models have been developed to study the pathophysiological changes in chronic lung diseases [6]. A miniaturized version of the CT, the microCT (μCT), is able to visualize lungs of small rodents [7]. Commercially available software is used to analyze μCT data, although to date these programs possess at least one of two major disadvantages. They either only provide semi-automated analysis (requiring additional time-consuming manual input but potentially captures the entire organ), or run fully automated but only capture aerated lung tissue, thereby missing important areas of pathology [8, 9].

Another approach to analyzing changes in lung structure for both humans and animal models is histological analysis. Clinical readouts regarding the status of lung tissue of a patient are based on a biopsy, a small fraction of the whole tissue considered representative [10, 11]. Here, we describe a novel approach for the analysis of μCT data from mice. We combine deep learning (DL) methods and “computational biopsies” of μCT data, by which we analyze not the entire lung but a representative volume of the lung. With the help of two neuronal networks we get information needed for a correct placing of a biopsy procedure within the left and right lung lobe. We show that this approach captures non-aerated areas and importantly, the calculated parameters correlate with outcome-related readouts, which include lung function parameters, the Ashcroft score (bleomycin model) and the mean linear intercept (MLI) of alveoli (elastase model).

## Methods

### Animals

Female or male C57BL/6 J mice aged 8–12 weeks were purchased from Charles River or Taconic. Groups of two to five mice were housed in individually ventilated cages at 22–25 °C, a humidity of 46–65% and 12-h day/night cycle. Animals received water and food ad libitum. Ethical approval for these studies was obtained from the regional governmental animal care and use office (Regierungspräsidium Tübingen, Germany, TVV 14–013-O, 16–030-G and 16–028-G).

### LPS exposure model

Female mice were exposed to 1 mg/ml aerosolized *Escherichia coli* lipopolysaccharide O55:B5 (LPS, Sigma Aldrich) in phosphate-buffered saline (PBS). Mice were placed in a LPS-preflooded Perspex box and whole body exposure was performed by administration of aerolized LPS by a nebulizer (Parimaster®). Mice were exposed to a continuous flow of LPS aerosol for 25 min, followed by 5 min conditioning after the aerosol was discontinued. Animals were analyzed 4 h after exposure.

### Elastase-induced emphysema model

Female mice received 0.2 U Elastase/animal (Sigma Aldrich) in PBS intratracheally (i.t.) under 3–4% isoflurane anesthesia once on day 0. Elastase administration was performed in a hanging position with a Vasofix-Braunüle 22G. Animals were analyzed 14 days after elastase administration.

### Bleomycin-induced fibrosis model

Male mice were instilled as described for the elastase model, using 0.45 mg/kg of bleomycin sulphate (Merck) in saline solution. Control mice received saline solution only. Mice were analyzed 21 days after bleomycin administration.

### Lung function acquisition

Mice were anesthetized using Narcoren (Boehringer Ingelheim) and lung function parameters were obtained using the Flexivent FX1 system (Scireq). Mice were ventilated with a tidal volume of 10 ml/kg at a frequency of 150 breaths/min. Each measurement was performed four times per animal.

### Histology

The left lung lobe was filled using 4% formalin solution (Sigma) and constant pressure (20 cm fluid column) for 20 min. Subsequently, lungs were embedded in paraffin for histological analysis (Masson trichrome staining). A pathologist performed the Ashcroft scoring [12] and MLI measurements were calculated using a proprietary image analysis solution based on Hsia and colleagues [13].

### μCT imaging procedure

Mice were first anesthetized using 3–4% isoflurane and, subsequently, lung imaging was performed using a Quantum FX μCT scanner (PerkinElmer Inc.). Cardiac gating strategy was performed as previously described [14]. The images were acquired at 90 kV, 80 μA and the lungs were scanned 360° for 4 min to capture the entire lung. An image stack of 512 slices with voxel size of 0.04 mm × 0.04 mm × 0.04 mm was generated.

### Processing and analysis of μCT images

Threshold-based, semi-automated segmentation and generation of lung densitometric data were done using Analyze 12 (AnalyzeDirect, Inc.).

The automated lung analysis application was developed using a commercially available machine vision software library (Halcon 19.5 Progress, MVTec Software GmbH), that offers a variety of pretrained networks, based on known (AlexNet and ResNet [15, 16]) and proprietary architectures for different purposes. Our application combines three deep learning methods: classification, object detection and segmentation. μCT-images are converted to byte format prior to labelling for training and for inference.

The first network sorts μCT images into classes with specific parts of the lung, which are: not lung tissue, only bronchi, top/front part, middle part, or bottom/rear part of lung. The training set has about 4000 images from up to 50 different μCT-stacks. We used images from animals of different ages and health conditions.

The second network is used for detecting landmarks in μCT images to allow positioning of the biopsy. All 1630 images in the training set show at least parts of a lung. In these images a rectangular box surrounding breast and spline bone was drawn and marked with a label.

In both cases image size is reduced to 224 × 224 pixel during preprocessing. Augmentation includes a random rotation around the middle from − 10 to 10 degree, a shift of up to 4 pixels in all directions and a brightness variation of max 5%. Training was started with a momentum of 0.9, a weight decay of 0.001, and a decaying learning rate from 0.001 to 0.00001 over 150 epochs.

A segmentation step was added for a visual control of the lungs. This is also done with a deep learning approach. The training samples for lung segmentation currently consist of more than 680 images and were labeled manually. Image size is kept at 512 × 512 pixel. Augmentation includes a random rotation around the middle from − 5 to 5 degrees, a shift of up to 10 pixels in all directions and a brightness variation of max 5%. Training was started with a momentum of 0.99, a weight decay of 0.001, and a decaying learning rate from 0.0005 to 0.00003 over 2500 epochs.

We used Tibco Spotfire 7.11 for data reduction and GraphPad Prism 8 for statistical calculations.

## Results

### Analysis of μCT images using analyze 12 is a semi-automated process

**Fig. 1:**
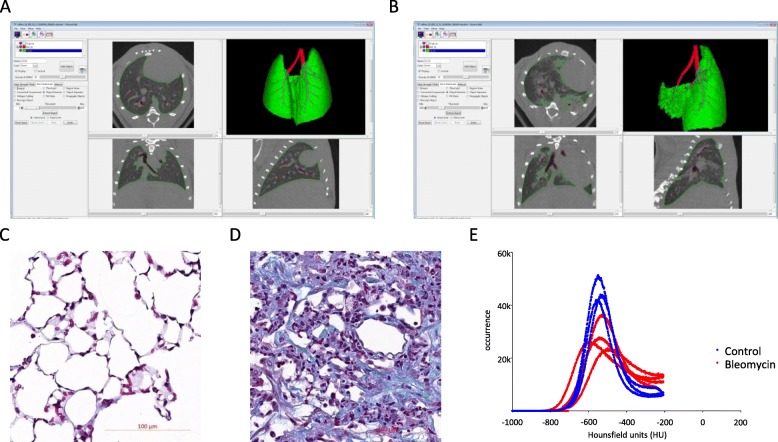
μCT *data analysis using a semi-automated program results in loss of highly dense tissue.* Threshold-based segmentation of (**a**) a healthy and (**b**) a fibrotic lung using the Analyze 12. Histological overview of (**c**) a control and (**d**) a fibrotic lung using Masson Trichrome staining (Magnification: 20x). **e** Densitometric histograms of lungs using threshold-based segmentation of control (blue) and fibrotic (red) lungs

Initially, we imaged bleomycin-treated mice and used Analyze 12 for segmentation and analysis of the μCT data. Images were generated in transversal, sagittal and vertical direction (Fig. 1a – control lung; Fig. 1b – fibrotic lung). Corresponding histological views for the same animals are provided in Fig. 1c and d (control and fibrotic lung, respectively). Bleomycin-treated mice showed an expected significant decrease in multiple lung function parameters (data not shown). The analysis of these μCT images started with the segmentation of a μCT stack into voxels belonging to airway and lung. It is possible to achieve a complete representation of airways and interstitial areas of the lung of healthy mice using thresholding and morphological operations (Fig. 1a). However, if the lungs are highly fibrotic, the fibrotic lung tissue will have the same intensity values for soft tissue organs like heart or liver. Thresholds set by the program for lung tissue usually have lower values than soft tissue and therefore highly fibrotic areas are not detectable in the semi-automated segmented lung volume (Fig. 1b). Because of this threshold, the intensity histograms have an upper limit at 1850 (corresponding to − 206 HU; Fig. 1e).

### Setup of a DL approach to automatically segment and analyze μCT images

**Fig. 2:**
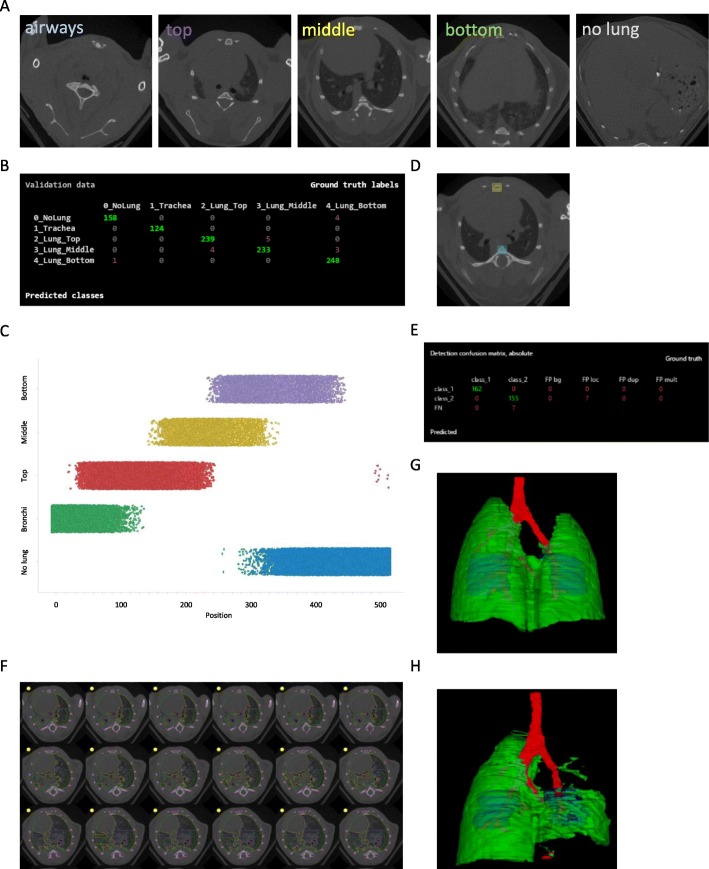
*Model development for analysis of* μCT *data using a deep-learning approach.***a** Example images for the five classes used for finding slices in the “middle of the lung” and (**b**) a confusion plot to illustrate the classification performance. **c** Plot of lung type class versus position in the μCT stack. **d** Example image for the detection of breast (yellow square) and spine bone (blue square) used to fit the “biopsy” ROIs inside the chest cavity and **e** a confusion plot to illustrate the detection performance. **f** Representative μCT images from class “middle of the lung” with segmented tissue using the deep learning approach and the fitted two “biopsy” ROIs. Segmented μCT stack with lung tissue (green), airways (red) and ROIs (blue) of (**g**) a control and (**h**) a severely fibrotic lung

We used a convolutional neuronal net for classification (based on GoogLeNet) of specific parts of the lung in the μCT images. We defined five classes: not lung tissue, only bronchi, top/front part, middle part, or bottom/rear part of lung (Fig. 2a). A confusion plot (Fig. 2b) shows the performance of the trained network.

The number of slices belonging to each lung part class and their location within each image stack varies (Fig. 2c). About 60 to 100 slices belong to the middle part of the lung (yellow dots in Fig. 2c).

Because of changes in animal position in the μCT scanner, the actual position of lung lobes in image stacks is not fixed. Landmarks like spline and breastbone (yellow and blue box, respectively; Fig. 2d) can be found with a neural network for object detection. A confusion plot (Fig. 2e) shows the performance of the trained network. With the coordinates from these two bones, we get information about size, translation, and rotation of the chest cavity in the image stack. This allows the positioning of two regions (green circles, Fig. 2f) in places where lung tissue is expected. This process is restricted to images belonging to class “middle part” of lung. The “biopsy” covers 14 ± 3% of lung volume.

We aimed to create a fully automated analysis algorithm for μCT stacks independent of segmentation success. Still, for a visual control of the lung status, the μCT stacks were segmented in a 2D slice–by-slice technique with the help of a convolutional neuronal net for segmentation (based on resnet-50 architecture). Segments were categorized in five classes (tissue, airways, bone, background, and ignore). The outline of the segmented lung and airway regions are used to generate a 3D representation for visual inspection of lung status. The generated 3D representation shows the biopsy voxels as blue objects within the lung lobes (Fig. 2g+h).

### Densitometry analysis of results from the DL approaches

**Fig. 3:**
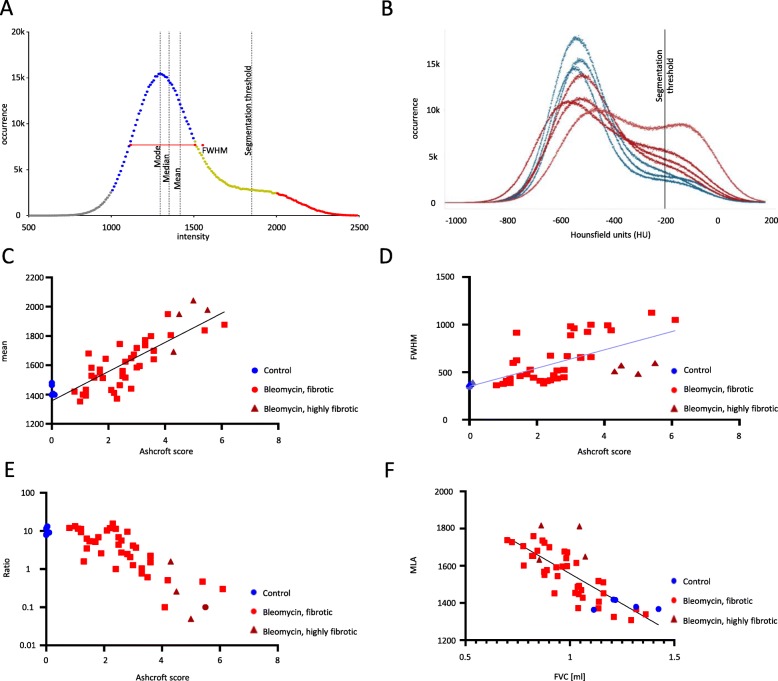
*μCT data analysis of fibrotic lungs using a novel deep learning approach.* (**a**) An example histogram from μCT data showing indexes used like FWHM and AUC areas (blue and red) for calculation of ratio. **b** Histogram of μCT data using a novel deep learning approach (control lungs – blue, fibrotic lungs – red). **c** Correlation of the intensity mean, (**d**) the FHWM and (**e**) the AUC ratio of the left lobe (AUC of normal tissue versus highly fibrotic tissue) versus the Ashcroft score. **f** Correlation of the FVC vs. the intensity mean of the whole lung

We obtained a segmented, but incomplete, lung volume using our application. All voxels in that volume were used to generate an intensity histogram to calculate values for mean or mean lung attenuation (MLA), median, mode, full width at half maximum (FWHM), skewness, and kurtosis (Fig. 3a). In addition, we obtained two biopsy volumes, one located in the left and one in the right lung. Here it is possible to calculate all features separately for each lung or summed up for both (Fig. 3b; histogram for both lungs). We excluded voxels from analysis that are segmented to airways.

### Results of DL tool correlate with clinically relevant parameters

Usually the left lung is used for histological examination. With our biopsy-based approach, we obtain μCT data for both lungs. Correlation of Ashcroft score versus voxel intensity mean (MLA) revealed an increase in Pearson correlation coefficient from *r* = 0.5789 for a threshold-based segmentation to *r* = 0.7477 for the biopsy-based analysis. The correlation increases to *r* = 0.8330 (*p* < 0.0001) when comparing only corresponding left lungs (Fig. 3c). The FWHM is frequently used for analysis and correlation with the Ashcroft score was significant (*r* = 0.6399, *p* < 0.0001; Fig. 3d). Marked in a darker color are data points from severely fibrotic lungs, which do not show a typical histogram shape and have more than one peak (Fig. 3b).

An alternative is the calculation of an area under the curve (AUC) ratio. We calculate AUC in the intensity range between 1000 and 1500 (normal tissue) and the intensity range between 2000 and 2500 (highly fibrotic tissue). This ratio is more sensitive than the MLA and a robust readout for fibrotic lungs (*r* = − 0.7164, *p* < 0.0001; Fig. 3e).

Calculated parameters from μCT such as the MLA also correlate with lung function parameters such as forced vital capacity (*r* = −0.79, *p* < 0.0001; Fig. 3f).

### DL tool is applicable to different murine models of lung disease

**Fig. 4:**
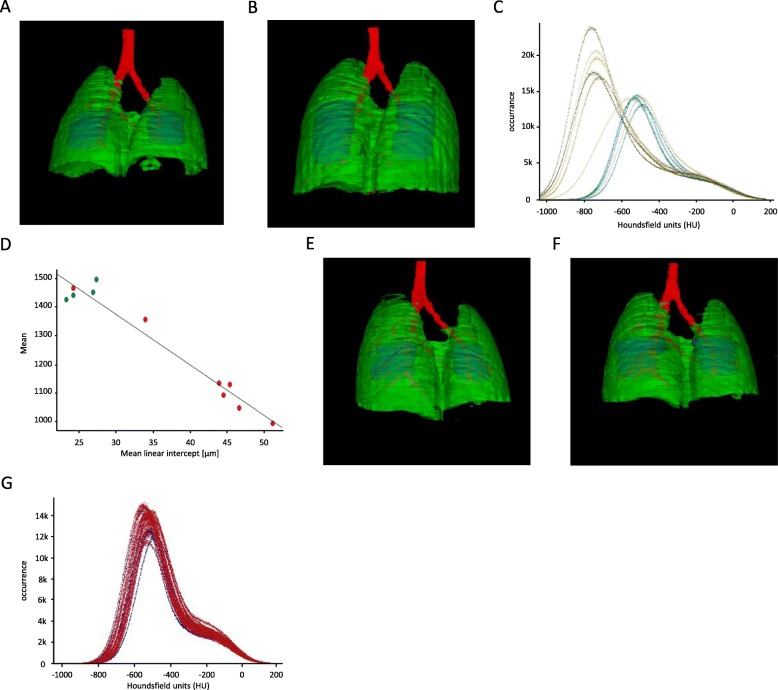
*Use of the automated analysis tool in other murine models of lung disease.***a-d** Results of μCT data analysis of a lung emphysema model. 3D renderings of (**a**) a control and (**b**) an elastase-treated lung. **c** Histogram of control (green) and elastase-treated (blue) lungs. **d** Correlation of mean linear intercept vs. mean intensity of the whole lung. **e-g** Results of μCT data analysis of a LPS model. 3D renderings of (**e**) a control and (**f**) a LPS-treated lung. **g** Densitometric histograms of control (blue) and LPS-treated (red) lungs

In a model of lung emphysema (Fig. 4a - healthy lung; Fig. 4b - emphysematic lung) the histograms of affected lungs are shifted to the left (Fig. 4c), representing a decrease in lung tissue density. Calculated μCT readout parameters from histological sections shows a correlation between MLI and MLA (Fig. 4d). Furthermore, the treatment of mice using LPS did not result in changes in the histogram compared to control animals (Fig. 4e – g).

## Discussion

The CT is one of the key tools in diagnosing patients with chronic lung diseases [17, 18]. Similarly, the μCT is used for the analysis of lung structure alterations in murine models of lung disease [19]. The tool presented here overcomes the major limitations of state-of-the-art software [7, 9]. It is fully automated and detects non-aerated tissue, delivers same-day results and is compatible with different mouse models of lung disease.

A fully automated and complete segmentation of whole lungs, especially in case of large injuries, is challenging. There is a wealth of human lung scan data but very little is reported for murine models [20, 21]. We utilized deep learning to help segment lung tissue, bronchus and bone of murine samples. This approach allows for inclusion of more fibrotic areas in the lung. The intensity histograms do not show an upper limit like the semi-automated histogram. With the biopsy volumes placed in both lungs, we are able to automatically include larger portions of non-aerated tissue and integrate these data into our analyses. The automated process also removes any inter-person variability in μCT analysis. Importantly, our results correlate with clinically relevant readouts. Results from the LPS model show that stimulation of murine lungs, which does not affect the stiffness of the lung at the time of analysis [22], are not detected as false-positive signal using our program.

In most cases, the intensity histograms of voxel intensities will have a non-Gaussian shape with one peak and calculation of skewness, kurtosis, and FWHM will give meaningful data. Lungs with strong fibrosis may have a second peak, generated from voxels representing dense tissue. This affects features that are based on peak position or shape. MLA is a more general and the most robust readout. An alternative for highly fibrotic lungs is the AUC ratio. It balances the appearance of a second peak in the histogram (loss of aerated tissue) and significantly correlates with the Ashcroft score. Our analyses were carried out with the raw intensity values of the voxels. The (linear) transformation from voxel intensity to Hounsfield units, not necessary for our analysis workflow, was only applied to the histogram graphs.

Although semi-automated analysis achieves similar correlation with the Ashcroft score (data not shown) when compared to our fully automated tool, the latter required less time. Manual curation of lung volume segmentation is an option to detect non-aerated tissue using semi-automated programs [23], but results in a significant increase of hands-on analysis time up to several hours per mouse. Our tool requires only raw data and a few minutes for the analysis of one image stack. Thus, it also facilitates longitudinal experiments, which use multiple low-dose μCT measurements in a single animal [9, 24].

The core of this labor-free automation is the use of “computational biopsies” from the lung tissue. Proper positioning of biopsy ROI is an essential part of the whole application. A simple location range in the μCT image stack is not appropriate, because of tolerances in positioning of the animal in the scanner and in morphological differences of lungs. For locating the lung in the μCT stack, we classified and sorted image slices into the appropriate lung part.

The slices classified as “middle part” of lung then contain two lung lobes. The location, orientation, and size of the lung lobes in the μCT images is also not fixed. To determine a correct place for the biopsy areas the location of spline bone and breastbone is found with a DL-based object detection. Their coordinates in space help in the determination of appropriate biopsy locations. The locations found in our “biopsies” resemble areas that are similar to where lung biopsies are taken from human patients [18, 25]. We tried networks of different architecture (AlexNet, GoogLeNet, ResNet-50) without noticeable difference in detection performance. However, the computational burden for training differs (from 35 to 80 min on an Nvidia GTX 1080ti GPU). First, a workflow for reading and analyzing images, and writing results has to be established. The integrated deep learning part is straightforward. Importantly, the tools required are not critical and open-source tools are also applicable. The image processing toolbox used for this application has full 3D object model capabilities but does not support voxel-based image processing and display. Therefore, the graphical representation of segmented lungs does not meet the normally expected quality criteria but serves its purpose in quality control and allows for a first impression of lung status. Additionally, radiation due to the μCT measurements of the animals can result in radiation-induced lung fibrosis. However, exposure time and radiation dosage were lower or equal to dosages previously reported to not induce lung fibrosis in mice [9, 26, 27]. Therefore, radiation applied to the animals will not affect the measured lung tissue densities.

## Conclusions

We here present a program, which allows for fully automated, timesaving μCT data analysis and the possibility to detect non-aerated lung tissue. Previously available and published programs only allowed for either automated analysis of μCT data without detecting highly stiff areas within the lung or required time-intense manual curation in order to include these areas. Areas of dense tissue are especially important when studying mechanisms involved in lung fibrosis as well as effects of potential antifibrotic strategies in murine models. Additionally, we could show that the here presented program also allows the analysis of μCT data of different murine models of lung disease. The here presented tool overcomes the disadvantages of available programs by automatically detecting non-aerated tissue and including these areas into the analysis.

## Data Availability

The datasets during and/or analysed during the current study available from the corresponding author on reasonable request.

## Abbreviations

μA: Micro-Ampere
μCT: Micro-computed tomography
CT: Computed tomography
DL: Deep learning
FWHM: full width at half maximum
i.t.: intratracheally
kg: kilogram
kV: kilo-Volt
LPS: Lipopolysaccharide
min: minute
ml: milliliter
MLA: Mean lung attenuation
MLI: Mean linear intercept
mm: millimeter
PBS: Phosphate-buffered saline
ROI: Regions of interest
